# Characteristics mode analysis and excitation of orthogonal modes on a single substrate for wideband IoT applications in the millimeter wave band

**DOI:** 10.1038/s41598-025-93578-1

**Published:** 2025-03-14

**Authors:** Ubaid Ullah, Slawomir Koziel, Anna Pietrenko-Dabrowsks

**Affiliations:** 1 Networks and Communication Engineering Department, Al Ain University, P. O. Box 112612, Abu Dhabi, UAE; 2 Engineering Optimization and Modeling Center of Reykjavik University, Reykjavik, Iceland; 3 Faculty of Electronics, Telecommunications and Informatics, Gdansk University of Technology, 80-233 Gdansk, Poland

**Keywords:** Millimeter-wave communication, EM-driven design, Circularly polarized antennas, Metamaterial, Unit-cell design, Engineering, Electrical and electronic engineering

## Abstract

This paper presents the design of a circularly polarized millimeter-wave (mm-wave) metasurface (MTS) antenna. The Characteristics Modes Analysis (CMA) is employed to examine various modes within the unit-cell design of the proposed metasurface. Based on a thorough analysis, two orthogonal TM modes with a broadside radiation pattern were identified. These modes were then simultaneously excited on a single substrate using simple coplanar waveguide (CPW) magnetic dipoles resulting in circular polarization (CP). Further, it has been demonstrated that the sense of polarization can be easily reconfigured for realizing multiple-input-multiple-output (MIMO) antenna with polarization diversity. Both the single MTS antenna and the MIMO design are characterized numerically and experimentally. The simulated and measured results show that impedance bandwidth (S11 ≤ − 10 dB) of the antenna is from 25 to 30.8 GHz. The axial ratio (AR) below 3 dB is from 26 to 31 GHz with a stable broadside radiation pattern. The proposed design features a low profile and simple geometry which is extremely appropriate for applications in the mm-wave band.

## Introduction

In the realm of contemporary wireless communication, including technologies just like the Internet of Things (IoT), 5G, 6G, and other prominent RF fields, a prevailing trend emerges: an escalating demand for heightened data rates in wireless communication. This surge in demand is accompanied by the utilization of significantly higher operating frequencies, often within the mm-wave range, and broader bandwidths than those employed in the past^1–3^. A challenge in long-distance mm-wave communication involves effectively managing the impact of multipath effects. In long-distance communication, electromagnetic waves must travel over extended distances, increasing the likelihood of the signal being obstructed by physical obstacles such as walls, trees, buildings, and so on. In the case of a linearly polarized wave, there is a heightened risk of complete data signal loss due to the increased severity of multipath effects and signal interference, especially in contemporary metropolitan areas^4,5^. Under such scenarios, circular polarization (CP) proves to be beneficial as it can meritoriously improve the signal by reducing multipath effects, absorption losses, signal interference, and signal attenuation. This capability ensures the retention of signal integrity.

Designing a high-performance, wideband circularly polarized (CP) antenna poses a significant challenge. Specifically, CP structures demand a sophisticated feeding circuit to generate two vector fields that are orthogonal and of the same magnitude^6^. Using a single-feed microstrip antenna is one of the straightforward approaches to achieve circular polarization (CP)^7^. However, this method results in a narrower 3 dB AR bandwidth which is usually 1.5%, primarily due to the excitation of only one mode. Various approaches have been proposed in the literature to improve the AR bandwidth. One of them is a multi-point-feeding technique, a frequently utilized technique for achieving wideband performances^7–9^. However, this method necessitates an intricate feeding circuit in comparison to the single-feed method. Consequently, considerable attention has been focused on enhancing the design of a single-feed antenna with improved AR and impedance bandwidth^10,11^. Furthermore, the electrical characteristics, especially AR, is generally very sensitive to the geometrical dimensions of the feeding structure. Even a slight alteration to the dimensions of the physical parameters or its position concerning the feeding point can lead to performance degradation. Therefore, streamlining the antenna’s overall geometry and the excitation circuit would enhance both the circuit precision and the fabrication yield. The MTS-based antenna is a type of application of the MTS that utilizes the resonant modes of a finite MTS for radiation. These MTS-based antenna designs feature low profiles, wide bandwidth, and easy implementation. Several MTS antennas have been proposed to meet specific requirements, including compact size (for size-confined applications), wideband capability, circular polarization, and low-profile applications^12–17^.

The CMA has proven effective in predicting the modal behaviors of metasurfaces, providing valuable physical insights into these antennas^18–21^. Consequently, the characteristics of metasurfaces can be anticipated by precisely adjusting the desired modes with the assistance of CMA^22^. Utilization of CMA for wideband CP MTS antennas has not been widely adopted, but its potential remains untapped. Few antennas with wideband characteristics were designed based on CMA analysis^22–25^. The drawback of these designs is the multilayer implementation with shorting pins is used for the excitation, their linear polarization, or narrow bandwidth. Only a limited number of single-layer MTS antennas have been proposed to achieve wideband CP radiation and maintain a low profile^21,22,26^. Furthermore, for Ka-band phased array systems, MTS-based designs offer significant advantages such as lightweight construction, accurate wavefront control, and simpler architectures that enable small and effective beamforming networks. Higher losses, stringent fabrication tolerances, and thermal management problems are some of the difficulties, especially in smaller designs where coupling, bandwidth limitations, and heat dissipation become more noticeable. Notwithstanding these challenges, MTS technology provides a convincing method for creating small and highly effective phased arrays at Ka-band by reducing the requirement for large phase shifters.

In this paper, an MTS-based wideband single-layer CP antenna for the mm-wave band is presented. A new octagonal star-shaped unit cell is designed, and its modal analysis is performed using CMA. Two orthogonal modes with broadside radiation and modal significance (MS) close to unity are then identified. This is followed by a CMA analysis of a 3 × 3 MTS to ensure the consistency of the modes. Simultaneous excitation of these two modes results in CP, therefore, a coplanar waveguide feeding in combination with magnetic dipoles (created using narrow slots) is used to excite the selected modes. The design is executed on a solitary substrate ensuring a compact, low-profile antenna. The numerical analysis of the design exhibits a wide impedance bandwidth from 25 to 30.8 GHz. The 3 dB AR of the antenna is from 26 to 31 GHz with a peak gain of 7.4 dBic in the broadside direction. The originality and the methodical contributions of the proposed work can be summarized as follows: (i) design of a new unit-cell and its uniform MTS design; (ii) achieving circular polarization using a simple feeding technique on a single substrate; (iii) demonstration of polarization reconfiguration by simple feed alteration; (iv) achieving wideband operation in terms of reflection coefficient and CP bandwidth, stable directional pattern, low mutual coupling; (v) realization of the aforementioned competitive performance using a low profile and easy-to-fabricate antenna structure.

## Metasurface design and analysis using CMA

**Fig. 1:**
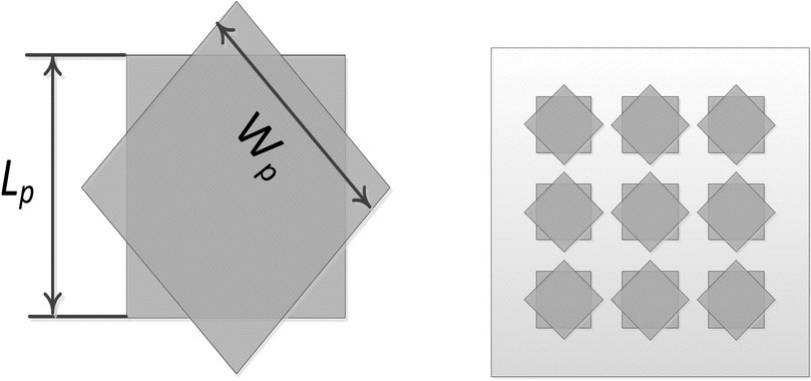
The proposed MTS design; Unit-Cell (left) with its geometrical parameters, and 3 × 3 MTS (right).

**Fig. 2:**
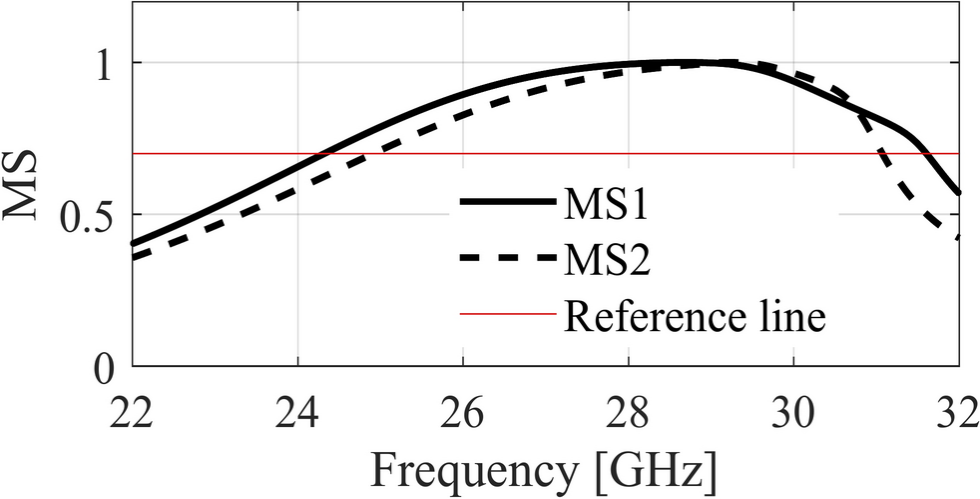
Modal significance of the proposed MTS.

**Fig. 3:**
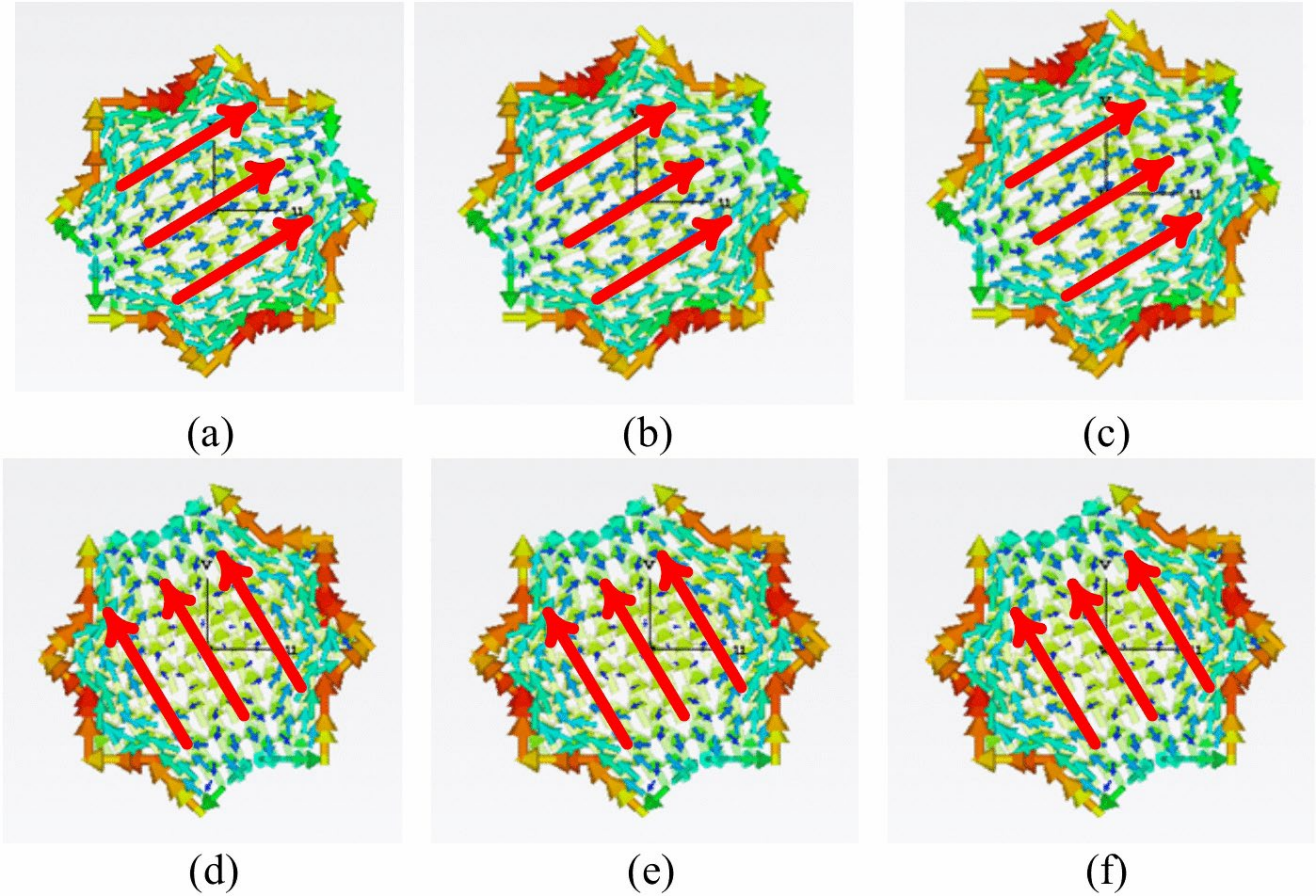
Surface current of the modes at different frequencies; mode 1 (top), mode 2 (bottom) (**a**) mode 1–27 GHz, (**b**) mode 1–29 GHz, (**c**) mode 1–31 GHz, (**d**) mode 2–27 GHz, (**e**) mode 2–29 GHz, (**f**) mode 2–31.

**Fig. 4:**
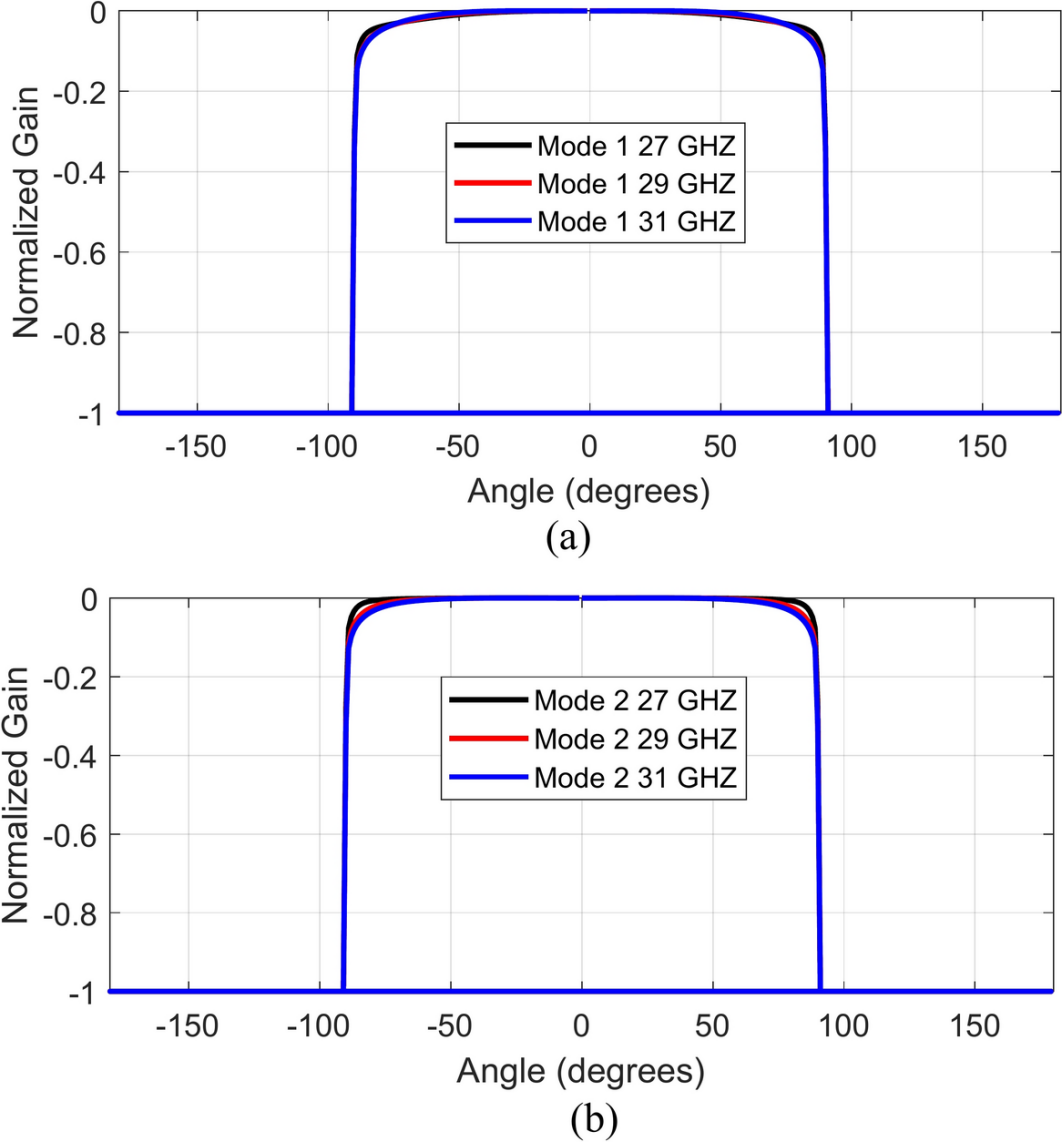
Radiation pattern of the two selected modes; (**a**) mode 1, (**b**) mode 2.

A unit-cell design and its respective 3 × 3 MTS of the proposed metasurface with its geometrical parameters are shown in Fig. 1. The unit-cell design has two metallic patches stacked diagonally on top of each other, forming an octagonal star-shaped structure. The first four modes of the unit cell are numerically characterized using commercially available computer simulation technology (CST) microwave studio software’s CMA analysis. However, only two orthogonal modes are selected for the proposed design. The significance of the chosen modes is shown in Fig. 2. Note that a mode is resonant when MS = 1 whereas it is non-resonant when its MS = 0. As $MS\ge 1/\sqrt{2}$ offers more effective radiation, therefore, a reference line is used at 0.707 to indicate that region^4,22^. Figure 2 clearly shows that the resonant frequencies of both modes are near 28 GHz. CMA’s dependence on frequency is a well-known factor^19^, leading to variations in both modal current and its respective radiation pattern. Hence, the surface current of mode 1 and mode 2 of the unit cell is shown at three different frequencies; i.e. 27 GHz, 29 GHz, and 31 GHz in Fig. 3. The figure reveals that both modes maintain a consistent modal current distribution across the frequency spectrum. Moreover, the surface current clearly indicate the phase difference between the two modes at all the frequency points. The combined vectors show the orthogonal field configuration of the modes with the peak current components for mode 1 along *M*_1(*x*,*y*)_ and mode 2 along *M*_2(*-x*,*y*)_. The orientation of the vector fields depicted offers substantial empirical evidence affirming the existence of orthogonal modes. Similarly, the Cartesian radiation patterns of both the modes is illustrated in Fig. 4 at the same frequencies. It is seen that both modes are radiating energy in the broadside direction at all frequencies. Therefore, simultaneous excitation of these modes results in the CP antenna with broadside radiation.

## Antenna design and analysis

### Antenna design

**Fig. 5:**
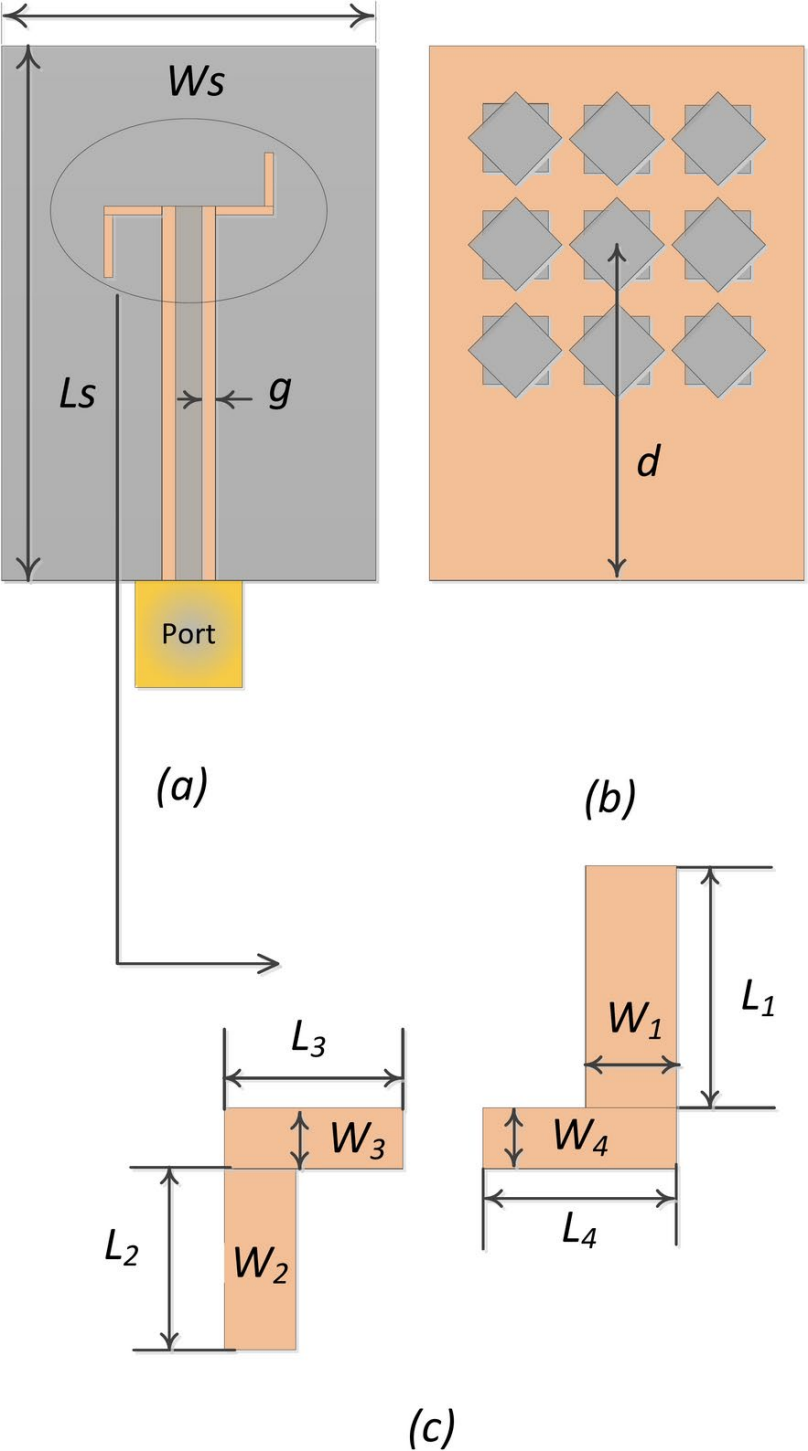
Geometry of the proposed single-layer MTS-based antenna; (**a**) coplanar waveguide (top view), (**b**) 3 × 3 MTS (back view). (**c**) Enlarged parameterized slots (magnetic dipoles).

**Fig. 6:**
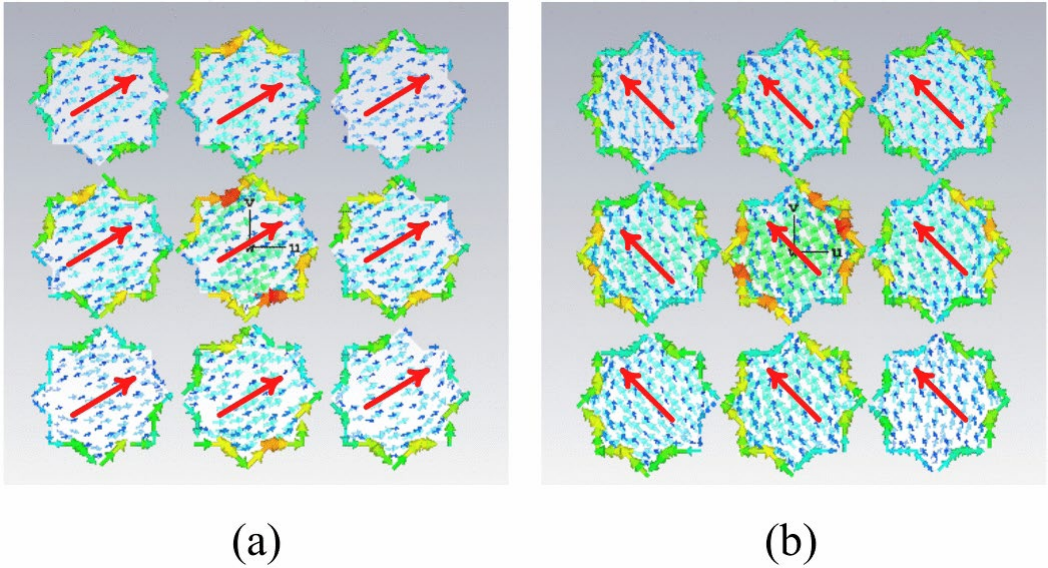
Surface current of the modes; (**a**) mode 1 MTS, (**b**) mode 2 MTS.

The proposed design is executed on a single Rogers RO4003 substrate with a dielectric permittivity *ε*_*r*_ of 3.38 with a thickness *h* = 0.813 mm (mm). A 50-Ω coplanar waveguide feeding is designed on top of the substrate, and two narrow slots are etched on either side of the feeding line to generate a magnetic current source. The coplanar gap between the feedline and the coplanar ground plane is fixed at *g* = 0.313 mm while the width of the feeding line is *W*_*m*_ = 1.05. The geometry of the antenna is illustrated in Fig. 5. The front view of the feeding configuration is shown in Fig. 6a, and the 3 × 3 MTS loaded at the back of the substrate is illustrated in Fig. 5b. Moreover, the enlarged parameterized geometry of the slots is depicted in Fig. 5c.

The dimensions of the slots are simultaneously optimized with the MTS radiator. To effectively excite the two orthogonal modes, the current sources are positioned at the point where the magnetic field strength of the modes is dominant. The parameter *d* shown in Fig. 5b is used to show the distance of the MTS from the port to the center of the MTS. It plays a crucial role in positioning the MTS on top of the magnetic current and ensuring maximum energy coupling. The open end of the microstrip line is shortened with the coplanar ground to avoid any radiation within the operating band from the feeding line. All the physical parameters of the antenna are concurrently optimized using a multi-stage optimization at the full-wave level. The final optimum geometrical parameters are listed in Table 1. All the physical values are reported in millimeters (mm).

**Table 1:** Proposed antenna: optimized parameters.

Parameter	Value (mm)	Parameter	Value (mm)	Parameter	Value (mm)
*L*_*s*_	21.999	*L*_*2*_	3.5567	*W*_4_	0.4030
*W*_*s*_	14.598	*W*_2_	0.6705	*L*_*p*_	2.0894
*D*	15.360	*L*_3_	2.2179	*W*_*p*_	1.9024
*L*_1_	3.7622	*W*_3_	0.2053	*W*_*m*_	1.0500
*W*_1_	0.5667	*L*_4_	1.9358	*L*_*m*_	13.7667

### Metasurface and its working principle

**Fig. 7:**
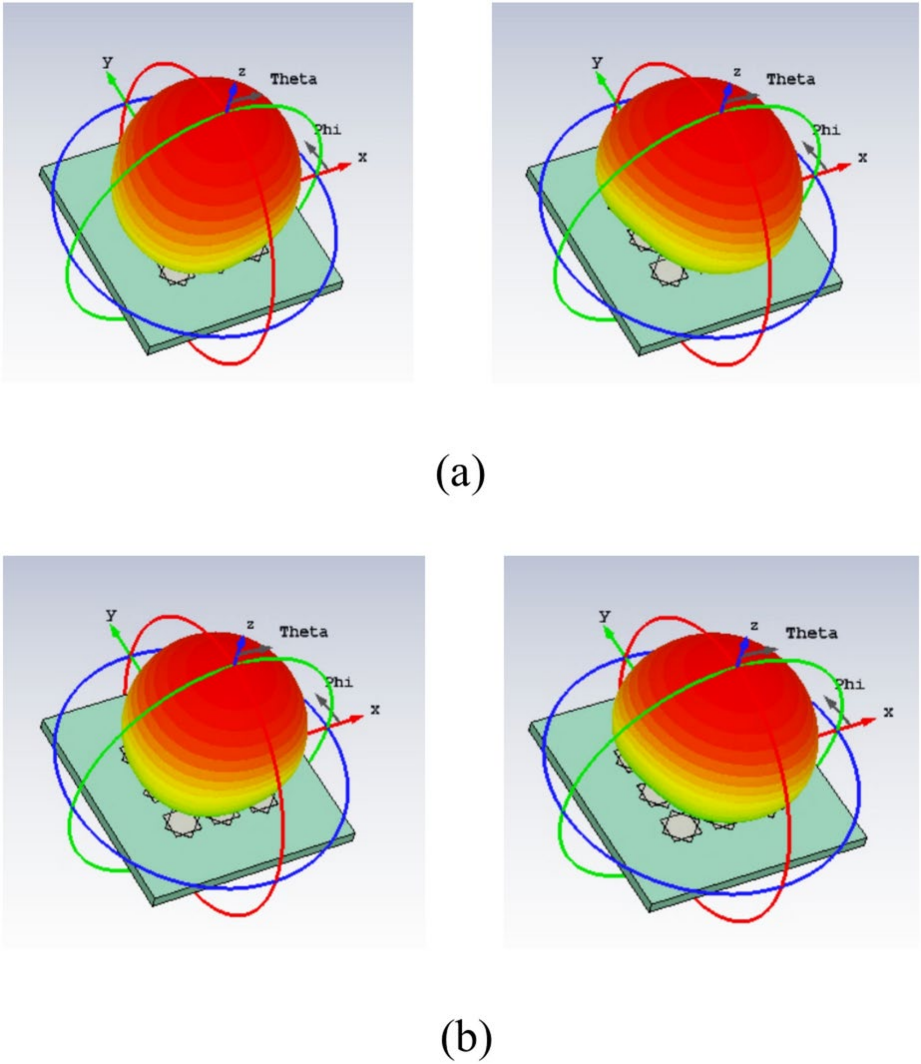
Radiation pattern of mode 1 (left) and mode 2 (right); (**a**) 27 GHz, (**b**) 30 GHz.

**Fig. 8:**
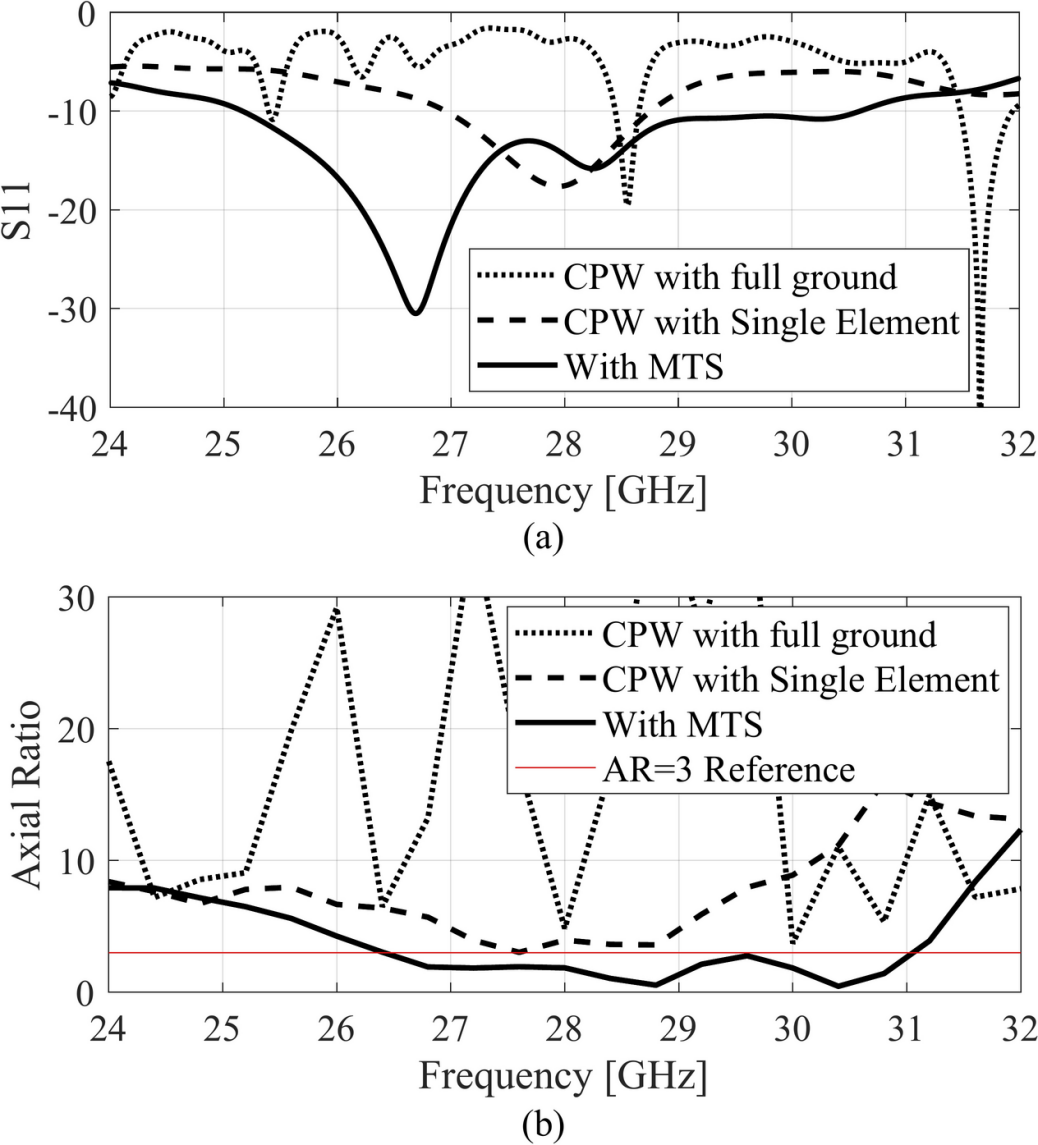
Analysis of the antenna in different configuration, (**a**) |S11|, (**b**) AR.

The working mechanism of the proposed MTS-based antenna is straightforward. The surface current of the two selected modes of the unit cell design and their radiation patterns are shown in Figs. 3 and 4. To further enhance our understanding of the entire MTS, the modal vector near fields is explored in conjunction with the current distribution. Figures 6 and 7 show the vector fields of the two modes on the 3 × 3 MTS and its corresponding farfield at two frequency points. This clearly indicates that the MTS retains the model behavior of the unit cell as well as the radiation pattern in the broadside direction. Moreover, the radiation patterns of the MTS are pointed in the broadside direction, which perfectly matches the unit cell behavior of the farfields. The two orthogonal modes identified using the CMA analysis are excited by the magnetic dipole currents created using the CPW slots. The selection of the feeding mechanism aims to excite the two modes by employing two E-field components with perpendicular polarizations linked to the aperture. The locality of the MTS is adjusted with reference to the excitation source so that the maximum current is aligned with the magnetic current of the slot and maximum energy is coupled to the MTS. The maximum modal currents are concentrated on the edges of the unit cell as well as the finite MTS, as seen in Figs. 3 and 6. Therefore, the L-shape slots are designed to ensure maximum energy coupling to the radiator. This method simultaneously excites both the orthogonal modes resulting in left-hand circular polarization (LHCP). This feeding approach ensures a physically simple, low-cost, low-profile, and involves a single substrate. To gain further insight, the performance of the proposed finite 3 × 3 MTS is compared with a single patch excited with the same feeding mechanism. Three different scenarios are studied. First, the CPW feeding with the slots is analyzed with a full ground plane at the bottom of the substrate. In the second phase, a single conventional patch design is excited using the magnetic dipoles of the CPW. These analysis are done to ensure the feedline done not radiate significantly within the frequency range, hence providing a baseline for assessing the effects of the MTS. Lastly, the proposed MTS is loaded, and its electrical characteristics are analyzed. Figure 8 compares the reflection coefficient and the AR of the three scenarios.

**Fig. 9:**
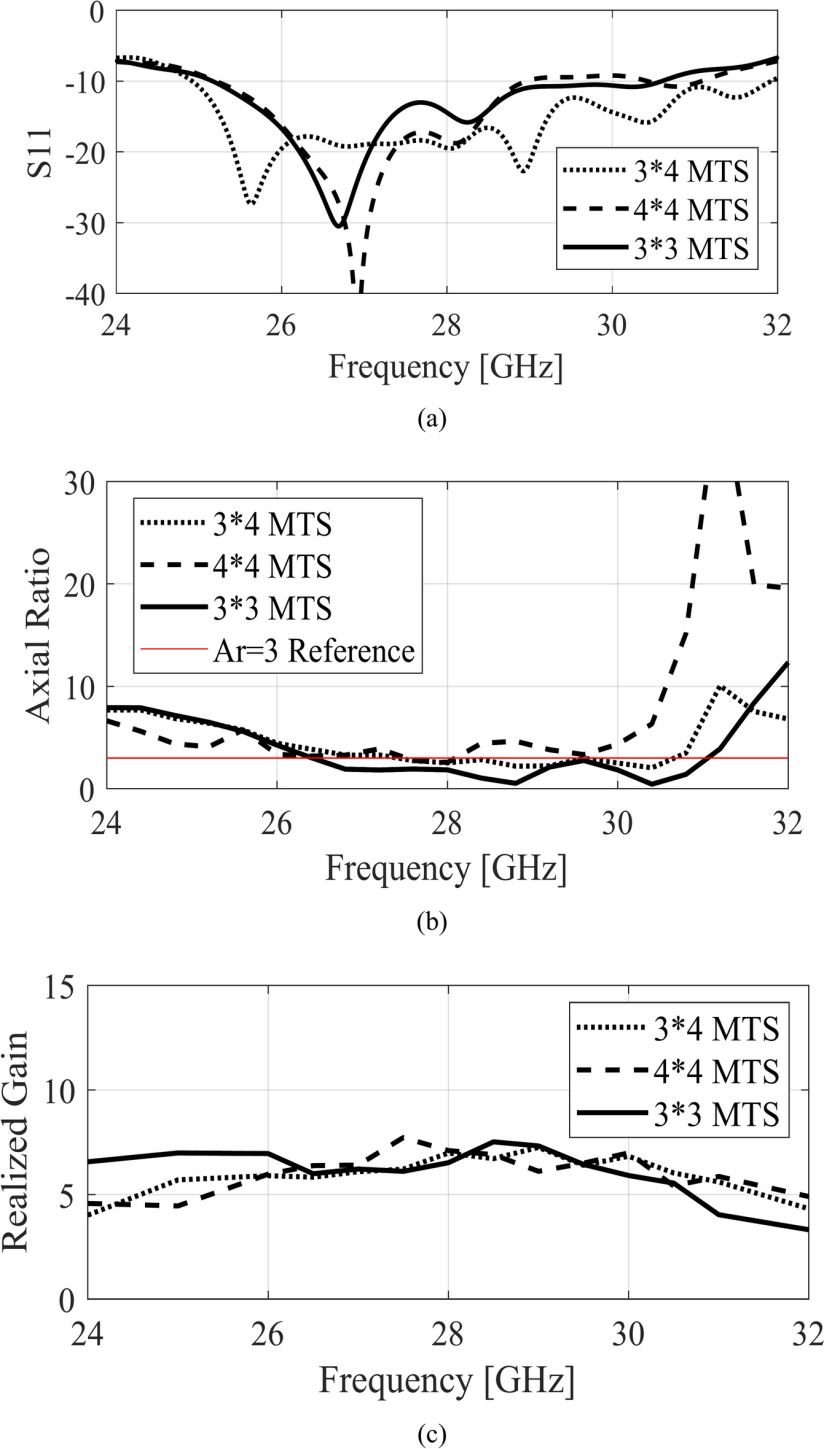
MTS with different numbers of unit cells, (**a**) *|S*_11_|, (**b**) AR, (**c**) realized gain.

The reflection coefficient of the CPW with the full bottom ground plane shows a very narrowband resonance near 28.5 GHz, as depicted in Fig. 8a. The AR response shown in Fig. 8b also indicates a poor AR for the feeding structure with a full ground plane. The impedance matching of the conventional patch design shows a roughly 2 GHz bandwidth from 27 to 29 GHz. The AR of the conventional patch design depicts a comparable characteristic to that of the MTS with approximately 2 GHz bandwidth nearing the 3 dB reference value. It is clearly observed that the proposed MTS features improved impedance bandwidth and AR bandwidth compared to the conventional patch. Furthermore, the effect of the number of MTS elements is studied in terms of impedance matching, AR, and realized gain, as illustrated in Fig. 9. The 3 × 4 configuration enhances the impedance bandwidth as the matching improves in the upper cut-off frequency. The effect on the AR bandwidth is marginal for this arrangement as the AR bandwidth almost remains the same as that of 3 × 3 MTS. The effect on the realized gain within the antenna’s operating band is also nominal due to limited energy coupling from the source to the MTS. A similar outcome is observed for the 4 × 4 configuration both in terms of impedance matching and AR. Overall, the realized gain of the three configurations remains almost the same. These results show that the feeding slots successfully stimulate the orthogonal modes of the 3 × 3 MTS, achieving an even distribution of energy owing to the symmetrical design of the structure.

### Multiple-input-multiple-output antenna design

**Fig. 10:**
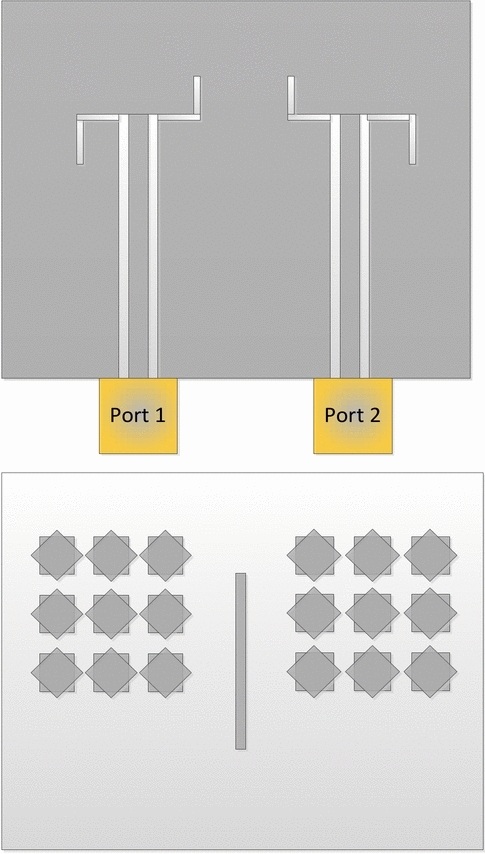
MIMO configuration of the proposed MTS-based antenna; feeding structure (top), MTS-radiator (bottom).

A single-layer MTS-based MIMO antenna operating in the 28 GHz mm-wave band holds promise for several applications including internet-of-things (IoT) devices, future smart cities, unmanned aerial vehicles, industrial automation, and healthcare telemetry. Therefore, the proposed MTS-based antenna is also implemented in a multiple-input-multiple-output (MIMO) configuration. The top and bottom of the MIMO antenna are shown in Fig. 10. To realize this design, the same substrate is extended in terms of parameter *W*_*s*_ to the right of the port resulting in (*W*_*s*_ = 29.197) for the MIMO design. The position of the narrow slots (magnetic dipoles) are switched with respect to the microstrip line as indicated in the top view of Fig. 10. All other dimensions, including the length of the feedline, the size of the slots, and the length of the coplanar ground plane are kept constant. The 3 × 3 MTS is loaded on top of the second port with the same dimension as the single antenna. The second port excites the two orthogonal modes with the opposite sense to that of the first port, resulting in right-hand CP (RHCP). To further improve the isolation between the two antennas on the common aperture, a simple passive metallic strip of width (*w* = 0.400 mm) and length (*L* = 9.1666) is inserted between the two MTS. The metallic strip, in conjunction with the simultaneous excitation of the two opposite senses of polarization, results in polarization diversity, a low envelope correlation coefficient (ECC), and high isolation between the two antennas. This arrangement enhances the antenna system’s performance by minimizing interference and improving signal quality. The MIMO antenna design is also fabricated and validated experimentally in terms of all major electrical characteristics.

## Experimental validation

### Return loss and axial ratio

**Fig. 11:**
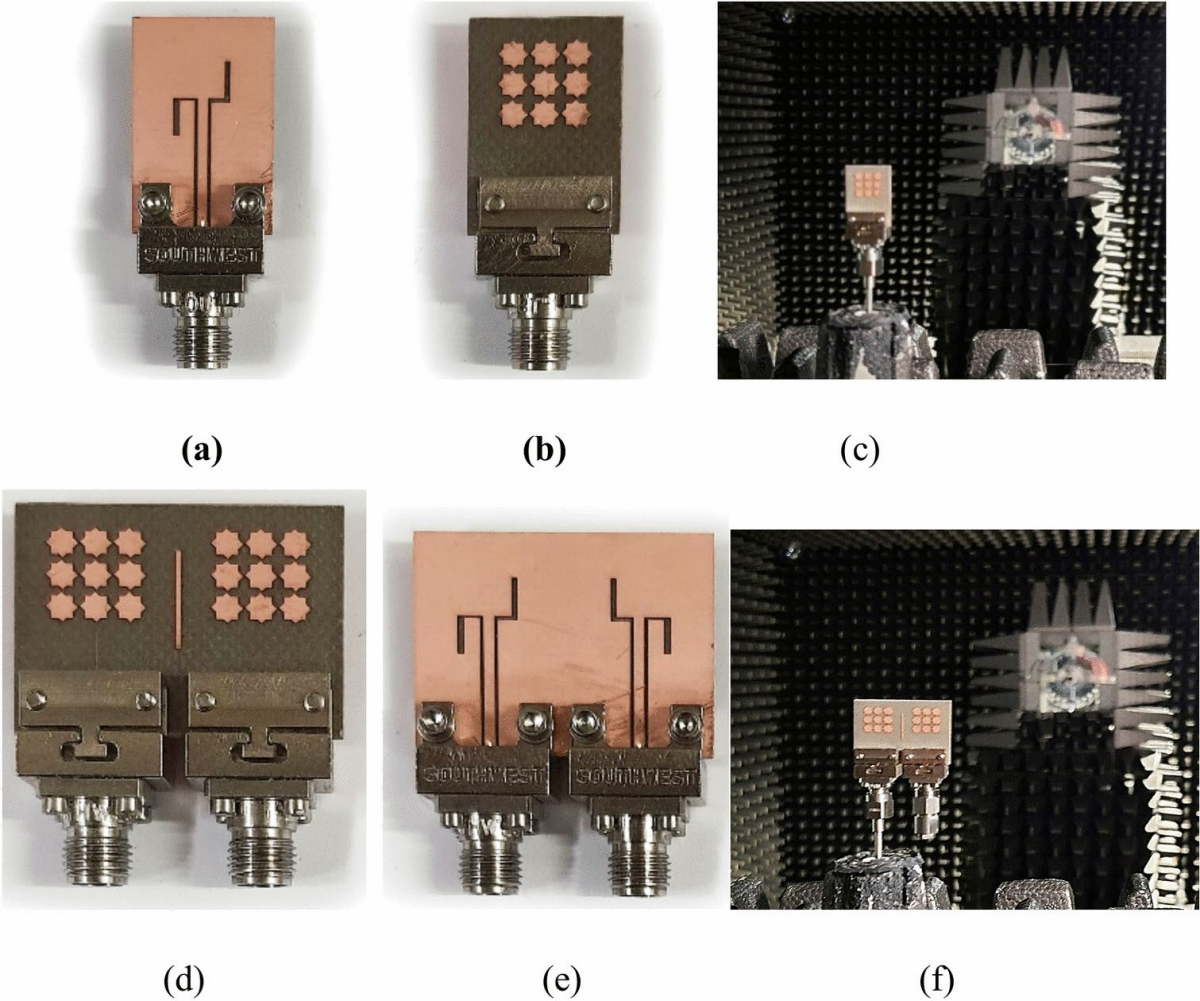
Photographs of the fabricated antenna prototype: (**a**) front, (**b**) back, (**c**) Experimental setup, (**d**) MIMO front, (**e**) MIMO back, (**f**) MIMO experimental setup.

**Fig. 12:**
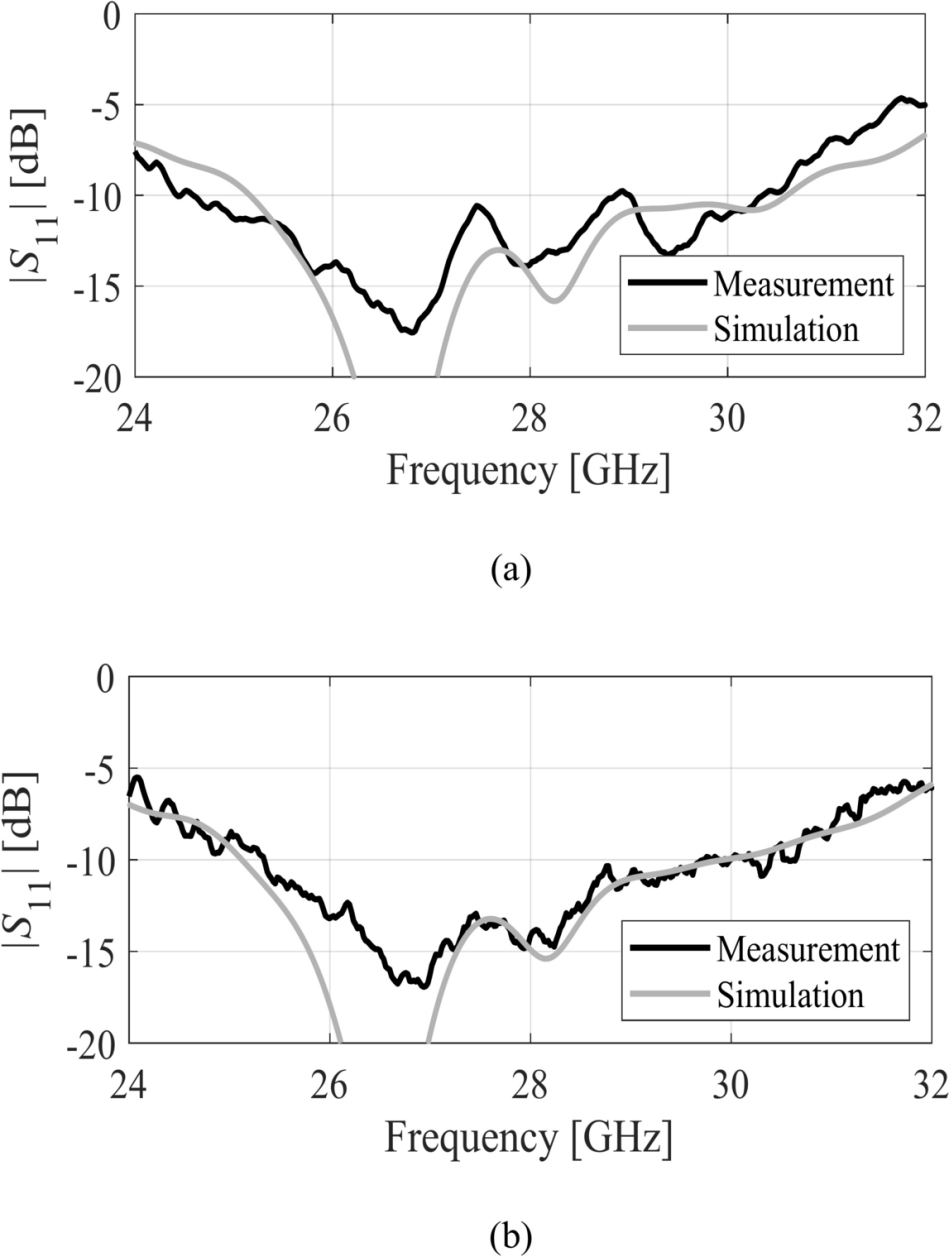
Simulated (gray) and measured (black) reflection coefficient |S11| of the proposed designs: (**a**) Single antenna, (**b**) MIMO antenna (Port 1).

The numerical evaluation of the proposed antenna, based on single-layer MTS technology, was conducted at the full-wave EM simulation level. To enhance the accuracy of the results, a Southwest end-launch connector identical to the one utilized in the experimental characterization was incorporated into the numerical model. All geometric parameters of the antenna were systematically optimized using a two stage optimization before experimental validation. The fabricated single antenna prototype, its experimental setup, the MIMO antenna, and its experimental setup in the fully calibrated anechoic chamber are depicted in Fig. 11. The experimental validation of the numerical results has been conducted at Reykjavik University, Iceland. The reflection coefficient plot of the single antenna is illustrated in Fig. 12a. The measured and simulated |S11| shows that the antenna has a wide impedance bandwidth of 6 GHz from 25 GHz to approximately 31 GHz.

**Fig. 13:**
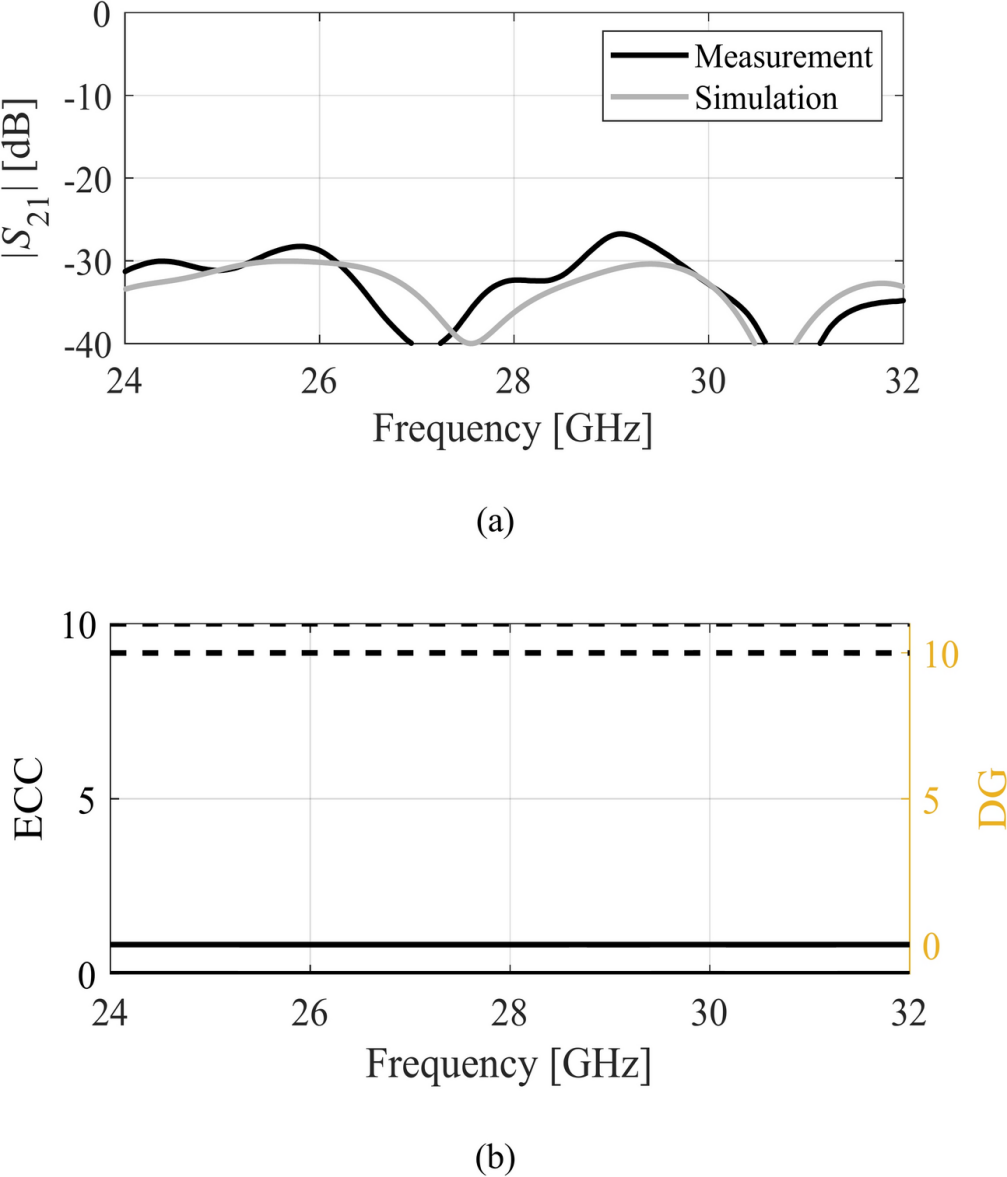
MIMO antenna characteristics, (**a**) simulated (gray) and measured (black) isolation of the MIMO antenna, (**b**) envelop correlation coefficient (—) and diversity gain (---).

Within this range, the impedance matching is below − 10 dB. The impedance matching of the MIMO antenna is shown in Fig. 12b. The impedance bandwidth of both the single element and the MIMO antenna is approximately 6 GHz, and the reflection coefficients are lower than − 10 dB within the mentioned band for both designs. The |S21| characteristic of the MIMO antenna is illustrated in Fig. 13a. The isolation of the antenna is greater than 25 dB without invoking any complex isolation technique.

The isolation value is attained due to a different sense of polarization of the two antennas, which results in polarization diversity. Furthermore, the envelop correlation coefficient (ECC) and diversity gain (DG) of the MIMO design are depicted in Fig. 13b. The solid line shows the ECC, which is almost zero reflecting a very low level of ECC. The latter can be attributed to the polarization diversity and high isolation. Similarly, the DG of the design is 10 dB which affirms the quality performance of the MIMO system.

**Fig. 14:**
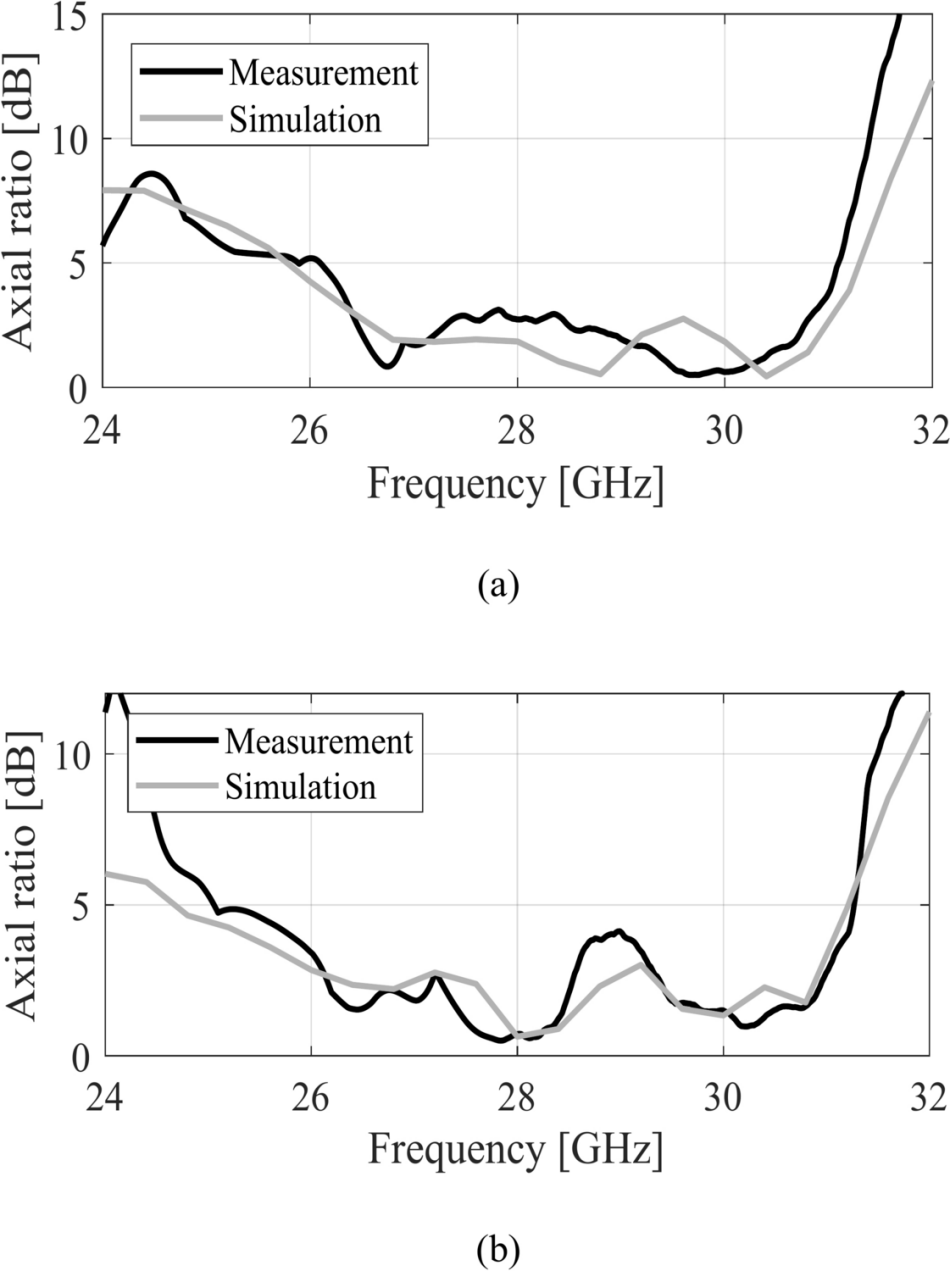
Simulated (gray) and measured (black) axial ratio of the MTS-based CP antenna, (**a**) single antenna, (**b**) MIMO antenna (Port 1).

Figure 14 shows the 3 dB axial ratio of the single MTS antenna. Both the measured and simulated values are below the 3 dB reference value in the frequency range between 26.3 and 31.1 GHz, indicating approximately 5 GHz CP bandwidth. It is important to highlight that the wideband CP response is achieved by exciting the two orthogonal modes using a simple feeding mechanism without involving complex multilayer geometries or excitation mechanisms. The MIMO design shows similar AR characteristics as indicated in Fig. 14b.

### Realized gain and radiation pattern

**Fig. 15:**
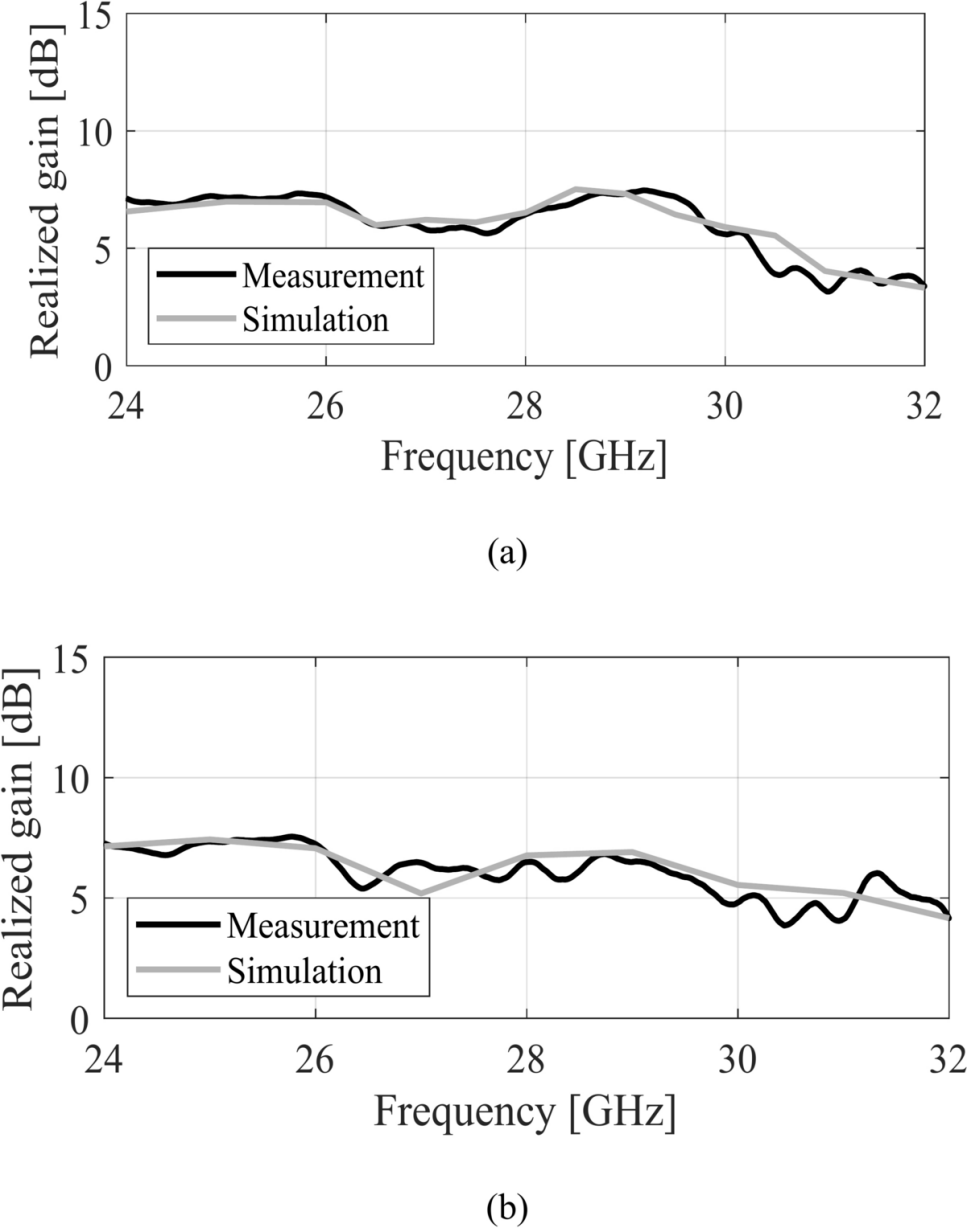
Simulated (gray) and measured (black) realized gain of the MTS-based CP antenna, (**a**) single antenna, (**b**) MIMO antenna (Port 1).

**Fig. 16:**
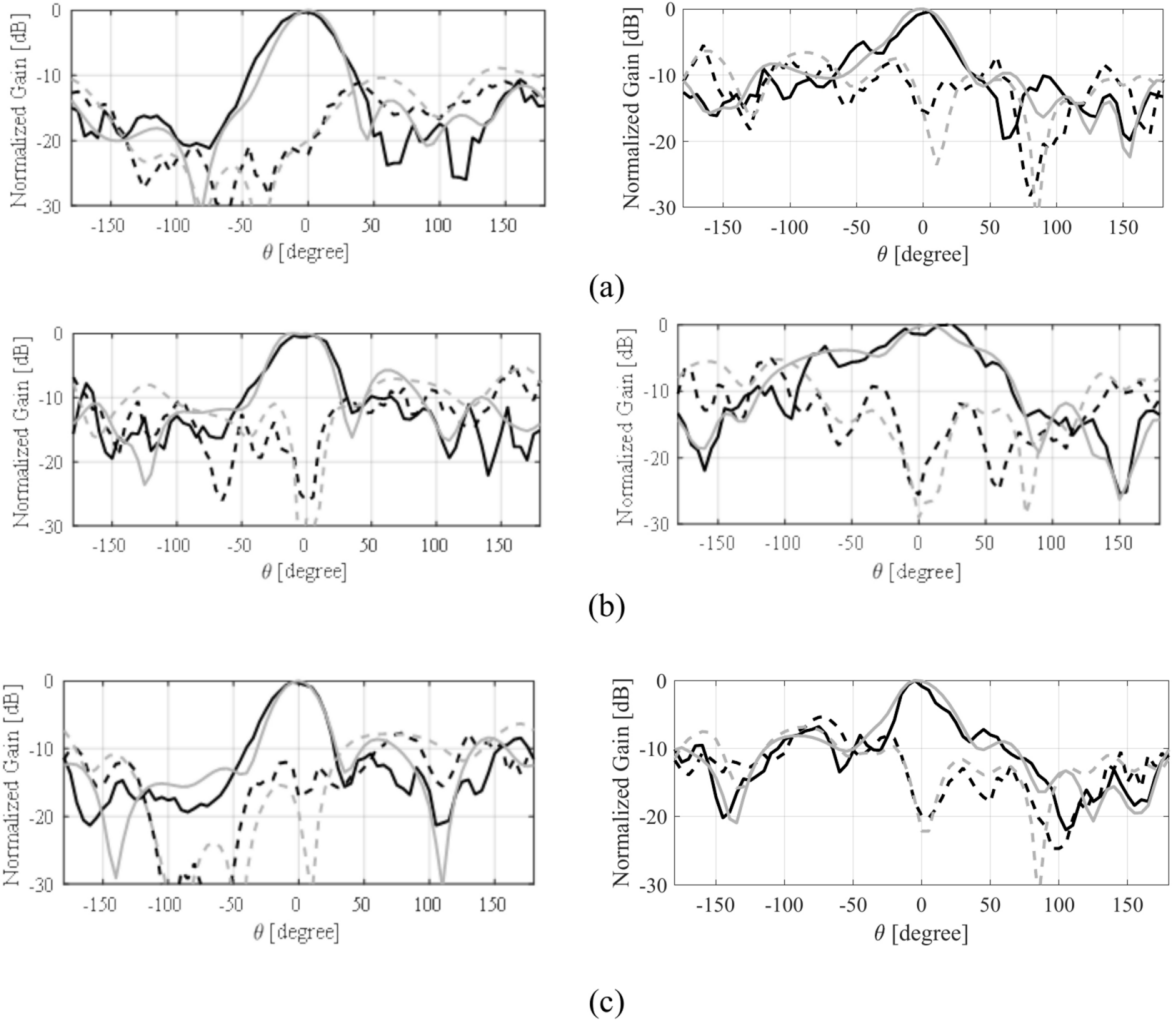
Simulated (gray) and measured (black) H-plane- (left) and E-plane (right) patterns for the single antenna at: (**a**) 27 GHz, (**b**) 29 GHz, (**c**) 31 GHz; LHCP (—), RHCP (---).

**Fig. 17:**
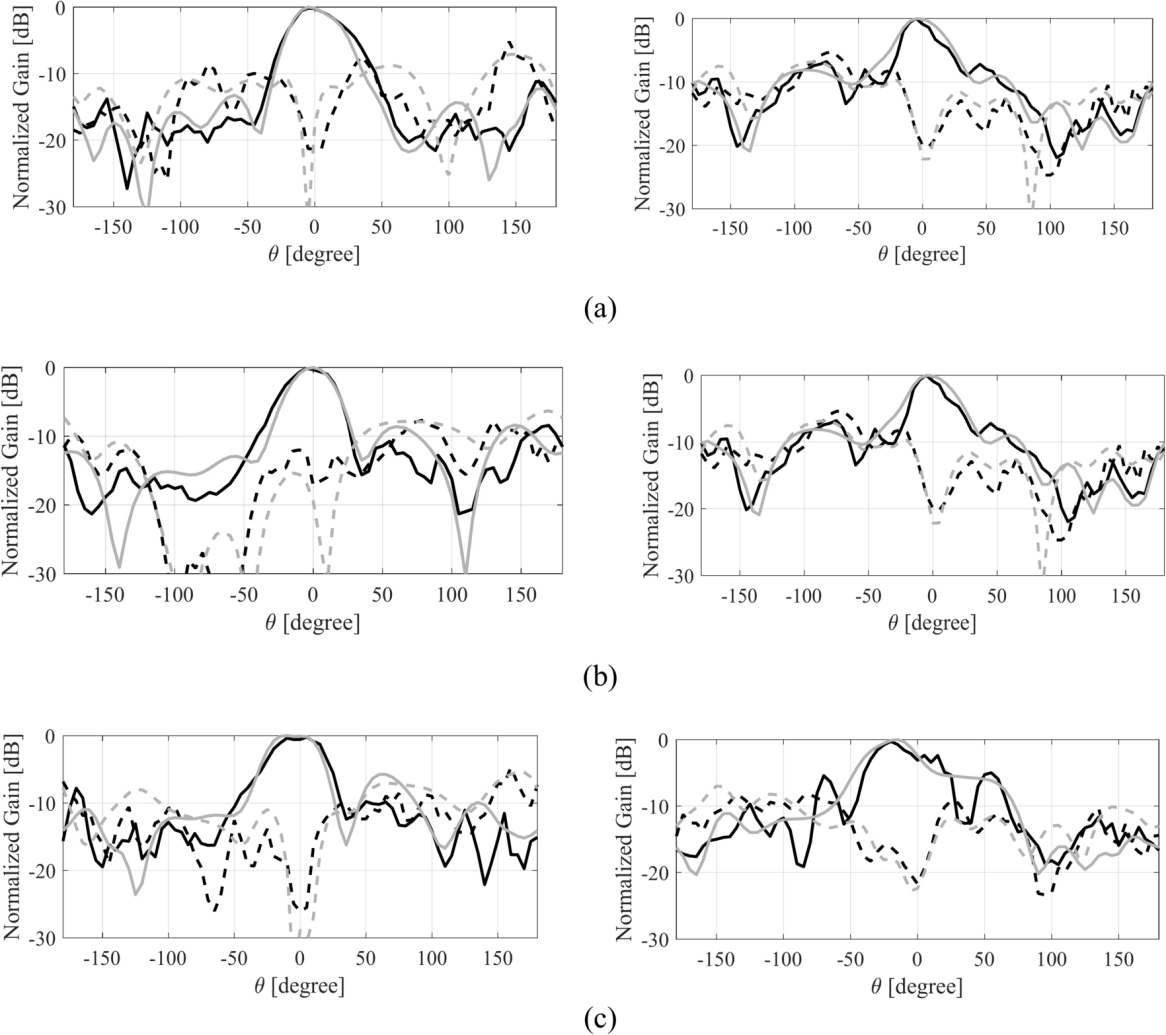
Simulated (gray) and measured (black) H-plane- (left) and E-plane (right) patterns for the MIMO antenna at: (**a**) 27 GHz, (**b**) 29 GHz, (**c**) 31 GHz; LHCP (—), RHCP (---).

**Fig. 18:**
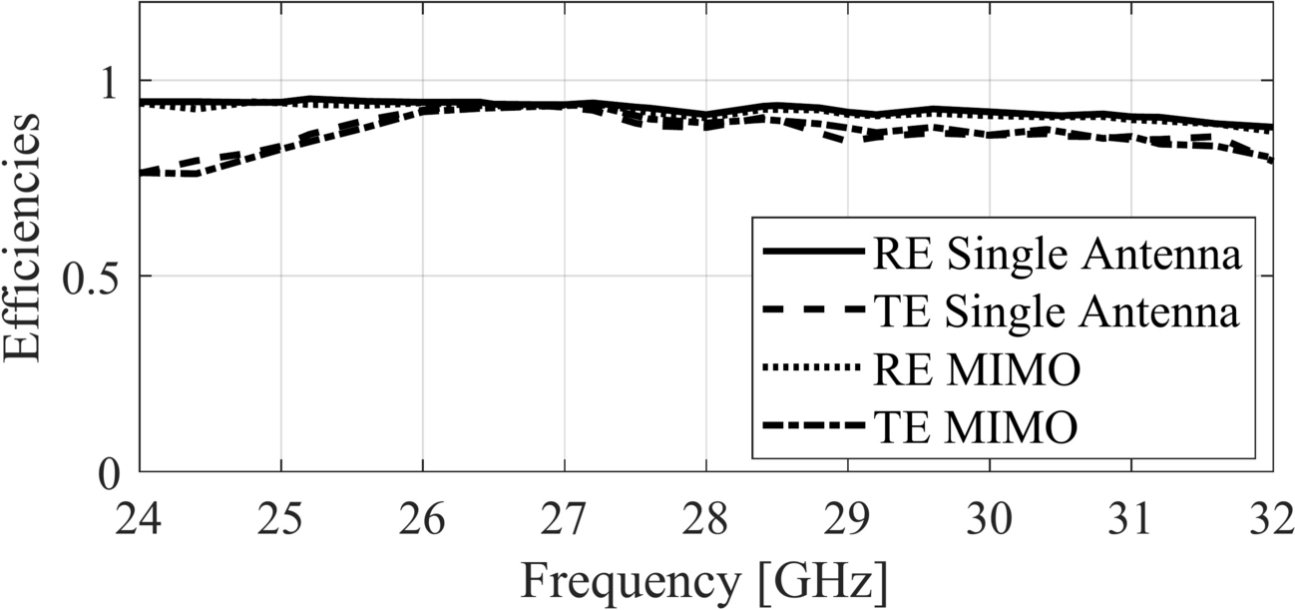
Radiation and total efficiencies of the proposed antenna.

The numerically analyzed and the experimental realized gain of both the antennas are depicted in Fig. 15. The experimental and the simulated realized gain of the antenna are in close agreement with each other. These results indicate a relatively constant in-band realized gain. The highest value of the gain is 7.5 dBic, while an approximately 6 dBic average realized gain is achieved in the operating band of the antenna. The MIMO antenna also exhibits a comparable realized gain with an average in-band value of about 6 dBic. The radiation pattern of the single antenna and the MIMO design are analyzed at three different frequencies. Figure 16 illustrates the H-plane and E-plane of the single MTS-based antenna. It is evident that the radiation pattern of the antenna closely resembles the pattern of the MTS, which showed a directional pattern in the broadside direction (see Fig. 7). The analysis of the radiation pattern at the selected frequency points confirms that the antenna is directionally radiating energy, thereby validating the simultaneous excitation of the two orthogonal modes (Fig. 17). Notably, the single antenna operates with left-hand circularly polarized (LHCP) fields as the co-polarization, whereas the right-hand circularly polarized (RHCP) fields represent the cross-polarized field. The cross-polar discrimination between the co-pol and cross-pol averages exceeds 20 dB in the antenna’s operating bandwidth, underscoring the high polarization purity of the proposed antenna. Furthermore, the radiation pattern of the MIMO antenna design is also analyzed at the same frequency points as that of the single antenna design. Figure 18 illustrates the radiation pattern of the MIMO antenna at multiple frequency points. A similar farfield pattern as that of the single antenna can be observed for the MIMO antenna. The cross-pol discrimination for the MIMO design is also appreciably high, which amounts to the value of approximately 20 dB in the whole antenna operating band.

## Antenna efficiency

The radiation efficiency (RE) and the total efficiency (TE) of the proposed single antenna and the MIMO design is depicted in Fig. 18. Note that RE reflects the ratio of the radiated power to the total input power. The average in-band RE of the single antenna is approximately 92.5% and the MIMO antenna is roughly 92.3%, which shows high conductivity of the antenna. Moreover, the TE (which includes both the RE and the impedance mismatch effects) is also illustrated in the same figure. The TE of the single antenna and the MIMO antenna is close to 86% averaged over the operating spectrum of the antennas, which demonstrates good impedance matching from the source to the MTS. Furthermore, the aperture efficiency of the proposed design is calculated using the peak gain in the broadside direction. The aperture efficiency of the antenna is approximately 51%, which is within a good range, considering the planar structure of the antenna.

## Benchmarking

The proposed MTS-based design has been compared to several state-of-the-art designs, considering a range of performance metrics. The design proposed in this study is benchmarked against references listed in Table 2. Comparison reveals that the proposed design surpasses the benchmark structures in several key areas, notably in impedance bandwidth, AR bandwidth, and the simplicity of the feed system. The design introduced in this paper features a significantly broader bandwidth relative to other designs, achieved through a simple feeding approach. The S11 bandwidth reported in Ref.^26^ is relatively wider, but the AR bandwidth is less than the proposed design, as well as the feeding mechanism utilized is the co-axial method, which adds to the complexity of the feeding. Furthermore, the presented design offers the added benefit of facilitating MIMO implementation, as demonstrated in the paper.

**Table 2:** Comparison table.

Refs.	AR BW (GHz)	S_11_ BW (%)	Number of layers	Substrate H (λ_o_), *ε*	Feeding complexity
^21^	LP	~ 4	Multi	(0.37, 3.5)	No
^27^	1.3	2.5	Multi	(0.28, 2.2)	Moderate
^28^	15	17.8	Multi	–	Yes
^29^	6	19	Multi	(0.07, 4.4)	No
^26^	16.8	~ 23	Single	(0.30, 2.2)	Yes
Proposed	18	~ 21.5	Single	(0.08, 3.38)	No

## Conclusion

In this paper, an innovative design of a single-layer circularly polarized antenna has been presented. The proposed design is based on a new finite MTS-based structure, excited using a simple CPW feeding mechanism. Before the antenna design implementation, a new unit cell design is comprehensively studied using CMA to identify orthogonal modes with similar radiation characteristics. Multiple modes of the unit cell design are analyzed, and two suitable modes are identified. Following this, a 3 × 3 MTS is analyzed using CMA to ensure mode consistency in the MTS. The radiation pattern of the unit cell design, as well as the 3 × 3 MTS are investigated to verify the pattern’s properties, especially its directivity. Subsequently, a CPW feeding mechanism is adopted to excite the MTS with magnetic dipoles created using narrow slits in the coplanar ground planes. The MTS is loaded at the back of the substrate and properly positioned to ensure strong coupling between the magnetic dipoles and the MTS. This results in simultaneous excitation of the two selected modes with broadside radiation. Furthermore, to show the design flexibility, the proposed MTS is implemented as an MIMO antenna with simple geometrical alteration of the feeding structure. All the electrical characteristics of the antenna are subsequently validated experimentally, showing a wideband performance in terms of reflection coefficient and axial ratio. The proposed designs are suitable for many space-constrained applications requiring fixed beams and wideband performance.

## Data Availability

All data generated or analysed during this study are included in this published article.
